# Supplementary material for: Distinct Thalamic and Frontal Neuroanatomical Substrates in Children with Familial vs. Non-Familial Attention-Deficit/Hyperactivity Disorder (ADHD)
Source: Brain Sci. 2022 Dec 26;13(1):46. doi: 10.3390/brainsci13010046 (PMC9856951; doi:10.3390/brainsci13010046)
Supplement: Supplementary file 1 [file brainsci-13-00046-s001.zip › brainsci-2061568-supplementary.pdf]

Full model analyses for thickness:

$y = \alpha + b_1ADHD + b_2Sex + b_3Age + b_4\ Handedness + b_5Race + b_6Education + B_7Puberty + B_8IQ + \gamma_i(MRI\ manufacturer) + \varepsilon$

Full model analyses for area and volume:

$y = \alpha + b_1ADHD + b_2Sex + b_3Age + b_4\ Handedness + b_5Race + b_6Education + B_7Puberty + B_8IQ + B_9ETIV$ (Estimated Total Intracranial Volume) $+ \gamma_i(MRI\ manufacturer) + \varepsilon$

Table S1: Full model results for gray matter neuroimaging measures between the control and ADHD groups.

Anatomical Location	Measure	TDC<br>Mean ± SD	ADHD<br>Mean ± SD	F-value	p-value
L. Cuneus	Surface Area	1676.39 ± 218.33	1633.97 ± 229.09	4.00	0.046
R. Middle Temporal	Surface Area	3997.17 ± 502.91	4011.51 ± 510.05	2.37	0.124

Means were compared using independent sample t-tests. SD: Standard Deviation. ADHD: Attention deficits/hyperactivity disorder group; TDC: Typically Developed Children.

Table S2: Full model results for gray matter neuroimaging measures between familial and non-familial subgroups of ADHD.

Anatomical Location	Measure	ADHD-NF<br>Mean ± SD	ADHD-F<br>Mean ± SD	F-value	p-value
R. Pars Orbitalis	Cortical Thickness	3.00 ± 0.17	2.95 ± 0.15	3.28	0.071
L. Inferior Temporal	Surface Area	3711.17 ± 498.36	3905.69 ± 579.72	7.58	0.006
L. Middle Temporal	Surface Area	3579.24 ± 446.97	3730.73 ± 540.07	5.32	0.022
R. Thalamus	Volume	7325.65 ± 649.59	7526.62 ± 770.62	3.027	0.083

Means were compared using independent sample t-tests. SD: Standard Deviation. ADHD-F: Familial ADHD. ADHD-NF: Non-familial ADHD.
